# The Combination Analysis Between *Bacillus thuringiensis* Sip1Ab Protein and Brush Border Membrane Vesicles in Midgut of *Colaphellus bowringi* Baly

**DOI:** 10.3389/fmicb.2021.802035

**Published:** 2022-02-18

**Authors:** Dengtian Cao, Changyixin Xiao, Qian Fu, Xinbo Liu, Rongmei Liu, Haitao Li, Jiguo Gao

**Affiliations:** ^1^College of Life Sciences, Northeast Agricultural University, Harbin, China; ^2^State Key Laboratory for Biology of Plant Diseases and Insect Pests, Institute of Plant Protection, Chinese Academy of Agricultural Sciences, Beijing, China

**Keywords:** *Colaphellus bowringi*, Sip, *Bacillus thuringiensis*, BBMV, insecticidal mechanism, competition binding assay

## Abstract

The secretory insecticidal protein Sip1Ab and crystal protein Cry8Ca from *Bacillus thuringiensis* (Bt) are widely recognized for their coleopteran insecticidal activities. It is worthwhile to investigate the insecticidal mechanisms of these two proteins against *Colaphellus bowringi* Baly, which is a serious pest of cruciferous vegetables in China and other Asian countries. To that end, the genes encoding the Sip1Ab and Cry8Ca proteins were amplified from the strain QZL38 genome, then expressed in *Escherichia coli*, after which bioassays were conducted in *C. bowringi* larvae. After feeding these two proteins, the histopathological changes in the midguts of *C. bowringi* larvae were observed using transmission electron microscopy (TEM), and the Brush Border Membrane Vesicle (BBMV) was extracted for competition binding assays. TEM showed that ingestion of Sip1Ab caused a significant reduction in growth of the larvae, disruption of midgut microvilli, and expansion of intercellular spaces. Competition binding assays demonstrated that Sip1Ab bound to *C. bowringi* BBMV with a high binding affinity. However, a mixture of the two proteins in equal proportions showed no significant difference in insecticidal activity from that of Sip1Ab. These results could provide a molecular basis for the application of Sip1Ab in coleopteran insect control and contribute to the study of the Sip1Ab insecticidal mechanism as well.

## Introduction

The cabbage beetle *Colaphellus bowringi* Baly (Coleoptera: Chrysomelidae) is a serious insect pest of crucifers in mountainous areas of China (Xue et al., 2002). Moreover, the application of chemical pesticides does not always effectively manage it (Gao et al., 2011; Shu et al., 2013).

*Bacillus thuringiensis* (Bt) is a Gram-positive bacterium belonging to the *Bacillus cereus* family. It can produce insecticidal proteins harmless to humans and the environment during growth and metabolism (Gómez et al., 2020). According to the protein location and secretion mode, these proteins are characterized as δ-endotoxins or exotoxins (Singh et al., 2021).

The δ-endotoxin is a protein with crystal structure encoded by the *crystal* (*cry*) and *cytolitic* (*cyt*) genes (Swiecicka et al., 2008). Current research shows that many *cry* genes have insecticidal activity against coleopteran pests, including *cry3*, *cry7*, *cry8*, *cry18*, *cry23*, *cry37*, and *cry43*. The *cry8* genes are currently the most studied, since they have specific insecticidal activity against many coleopteran insects, for example, the beetle family, the leaf beetle family, the weevil family, and so on (Jiang et al., 2017). In 1994, Beard et al. cloned a new insecticidal gene named *cry8ca1* from a *B. thuringiensis* strain with high toxicity to coleopteran beetles for the first time (Beard et al., 2001). On the basis of the established PCR-RFLP identification system of *cry8* genes, Liu and co-workers have cloned a novel *cry8C* gene from strain HBF-1, designated *cry8ca2* (AY518201) (Liu et al., 2004). Wu’s Laboratory has established a purification method for the Cry8Ca2 active toxin, and bioassay results have suggested that the insecticidal protein had good activity against the larvae of *Anomala corpulenta* and *Anomala exoleta* Fald (Wu et al., 2007). To date, a series of Cry8 proteins which contain about 1,200 amino acids, with the molecular weight of approximately 130 kDa, have been identified to have the high insecticidal activity to coleopteran pests (Noguera and Ibarra, 2010).

Exotoxin is a metabolite which is secreted outside the cell during metabolism of Bt, such as Vegetative insecticidal protein (Vip) and Secreted insecticidal protein (Sip) (Palma et al., 2014). It is now well established from a number of studies that Sip has the insecticidal activity against coleopteran larvae. Donovan et al. (2006) reported that Bt strain EG2158 secreted the coleopteran active protein Sip1Aa, which had insecticidal activity against larvae of the potato beetle (*Leptinotarsa decemlineata*), the southern corn rootworm (*Diabrotica undecimpunctata howardi*), and the western corn rootworm (*Diabrotica virgifera virgifera*). The gene encoding Sip1Aa, which has been reported as the first member of Bt secreted proteins having activity against coleopteran larvae, was 1,104 bp in length and encoded a polypeptide of 367 amino acids (Donovan et al., 2006). In 2012, Liu and co-workers successfully cloned and identified another *sip* gene from strain QZL26 encoding a 39 kDa protein, which shared a 91.83% identity with Sip1Aa (Liu et al., 2012). In 2015, our laboratory identified a *sip* gene from strain DQ89, which was 1,095 bp and encoded a protein of 364 amino acids with a calculated mass of 42 kDa (Zhang et al., 2015). The protein expressed by this gene showed high toxicity to *C. bowringi* with a lethal concentration 50 (LC_50_) of 1.542 μg/ml (Zhang et al., 2015). Sha et al. (2018) showed that Bt strain QZL38 contained a new *sip* gene designated as *sip1Ab* from 540 soil samples, which contained an open reading frame of 1,095 bp encoding a 364 amino acid polypeptide. The results of bioassays of four truncated mutant proteins indicated that the truncated protein SipA, from which the signal peptide (the first 30 amino acids) was removed, showed insecticidal activity against *C. bowringi*, with an LC_50_ of 1.078 μg/ml and showed no significant differences compared with Sip (Sha et al., 2018).

In previous studies, many Bt insecticidal proteins demonstrated synergistic or antagonistic effects (Lemes et al., 2017). Some studies have found that when Cry1Aa and Cry1Ac were mixed in various proportions, their insecticidal activities against *Lymantria dispar* varied. The insecticidal activity was increased by 3.8 times when the proteins were mixed in a proportion of 1:1, and it increased by 7.3 times when mixed in proportion of 1:2. However, a mixture of Cry1Aa and Cry1Ab showed antagonism (Xue et al., 2005). Moreover, a combination of Cry6Aa and Cry55Aa had significant synergistic toxicity to *Meloidogyne incognita*, and the insecticidal activity of these two proteins was increased five times when they were combined in a ratio of 1:1 (Peng et al., 2011). Additionally, in a study of the toxicity of Cry1 and Vip3A to *Diatraea saccharalis*, the proteins did not interact, but Cry1Ab and Cry1Fa had synergistic effects with Cry1Ca (Pavani, 2013).

Unlike Sip, the insecticidal mechanisms of the Cry and Vip toxins have been studied extensively. A study of the insecticidal mechanism of Cry toxins found that Cry was hydrolyzed and activated by a protease in the midgut of the target insect. The intestinal epithelium cell membrane was destroyed after the Cry toxins bound to a specific receptor, which led to the death of the insect pests (Deist et al., 2014). For the Vips, it has been found that Vip3Aa could bind to the intestinal cells of insects, then become activated by the alkaline midgut fluid, leading to cell fragmentation *via* binding to specific proteins in the midgut cells (Zhang et al., 2018). However, Wang et al. (2020) have indicated that the receptor for Sip might be located in the midgut of the *C. bowringi* larvae.

In previous research in our laboratory, it was found that strain QZL38 contained both the *cry8Ca* and *sip1Ab* genes (unpublished data), both of which may have synergistic or antagonistic effects. Given that, the *sip1Ab* and *cry8Ca* genes were cloned using the genome of QZL38 as a template, and the corresponding proteins were induced and expressed. By adding a mixture of the proteins Sip and Cry, changes in the midgut of *C. bowringi* larvae were observed under the electron microscope and compared with the group in which Sip alone was added. Competitive binding assays were carried out for further verification. One of the purposes of this investigation was the exploration of the relationship between Sip1Ab and the midgut of *C. bowringi* larvae; another was to verify whether Cry8Ca had a synergistic effect with Sip. This study indicates new directions for further research of protein interactions, and new resources for the prevention and control of coleopteran pests. It is hoped that this research will contribute to a deeper understanding of the insecticidal mechanism of the Sip toxin.

## Materials and Methods

### Construction of Expression Vector

The genomic DNA of strain QZL38 was extracted by using the EasyPure^®^ Bacteria Genomic DNA Kit (TransGen Biotech, Beijing, China) according to the manufacturer’s protocol and quantified using a Nano Photometer P300 (Implen, Inc., Munich, Germany).

The *sip1Ab* (KP231523.1) and *cry8Ca* (GU270856.1) genes were amplified by polymerase chain reaction (PCR) from the QZL38 genomic DNA sample. KOD -Plus- DNA Polymerase (Toyobo Co., Ltd., Osaka, Japan) was used for all PCR reactions. The reaction system consisted of 1× -KOD- Plus buffer, 0.2 mM of dNTPs, 1 μM of MgSO_4_, 0.3 μM of primer (for each), 200 ng of genomic DNA, and 1 U -KOD- Plus polymerase with a supplement of ddH_2_O up to a total volume of 50 μl. Amplification was performed for 35 cycles after pre-denaturation at 95°C for 2 min. Cycling conditions were set to denaturation at 95°C for 15 s, annealing at 59°C for 30 s, and extension at 67°C for 90 s. The amplification products were analyzed by electrophoresis on a 1% (w/v) agarose gel and purified using an Axygen^®^ DNA Gel Extraction Kit (Axygen Biosciences, Union City, CA, United States) following the manufacturer’s instructions. The DNA sequences of the oligonucleotides used in this study are listed in Table 1.

**TABLE 1:** Primers used in this study.

Name	Sequence (5′→3′)
cry8CaF	GTGGTGGTGGTGGTGCTCGAGATGAGTCCAAATAATCAAAATGAGTATG
cry8CaR	CCGAATTCGAGCTCCGTCGACCTCTTCTTCTAACACGAGTTCTACACTTT
sipF	GTGGTGGTGGTGGTGCTCGAGGCAGAAACCAAGTCGCCAAA
sipR	CCGAATTCGAGCTCCGTCGACATTTCCACTTAAAATCTTTGTTTGAACA

*Underlined nucleotides are the homology arms to pET21b.*

Restriction digestion was performed on pET21b at *Xho* I and *Sal* I sites to obtain a linearized plasmid. The insert fragment and the linearized pET21b were blunted by using a ClonExpress™ II One Step Cloning Kit (Vazyme, Nanjing, China). The recombinant plasmids were then transformed into *Escherichia coli* DH5α using the heat-shock method, and the recombinant strains were incubated on solid LB medium (containing ampicillin) at 37°C for 12–16 h. Verification of successfully constructed plasmids was performed *via* colony PCR and sequencing.

### Heterologous Expression of Sip1Ab and Cry8Ca

The recombinant plasmids were extracted using an Axygen^®^ Plasmid Miniprep Kit. A second transformation was carried out in *E. coli* BL21 (DE3) using the same method as above. Plasmid pET21b without an inserted fragment was used as a control in downstream experiments. The obtained transformants were inoculated into 5 ml of LB medium supplemented with ampicillin and pre-cultured overnight at 37°C, shaking at 220 rpm. These liquid cultures were then inoculated with 1% (v/v) into a 500 ml flask containing 100 ml of LB medium supplemented with ampicillin and incubated at 37°C, 220 rpm to an optical density at 600 nm (OD_600_) of 0.6. For inducing expression, isopropyl-β-D-thiogalactopyranoside (IPTG) was added into the cultures to a final concentration of 0.5 mM. After induction for 16 h at 16°C, 160 rpm, cells were harvested by centrifugation at 8,000 × *g* for 5 min at 4°C and washed at least twice with 10 mM phosphate buffered saline (PBS, pH 7.4). The cells were disrupted by ultrasonic treatment in an ice-water mixture for 3 s pulses for 10 min with 5 s intervals. Debris and unbroken cells were removed by centrifuging twice at 12,000 × *g* for 15 min at 4°C. The supernatant was filtered through a 0.22 μm filter and purified using a Ni-NTA Fast Start Kit (Qiagen, Hilden, Germany) according to the instructions in the manual.

Samples of the purified products were mixed with 2× SDS loading buffer [100 mM Tris–HCl pH 6.8, 200 mM β-mercaptoethanol, 4% (w/v) SDS, 2% (w/v) bromophenol blue, and 20% (v/v) Glycerol], immersed in a boiling water bath for 10 min, and centrifuged at 10,000 × *g* for 10 min. The supernatant was collected for protein separation using SDS-PAGE and visualized with a Coomassie blue stain. The protein concentration was determined using bovine serum albumin (BSA) as a standard following the user manual of the BSA Protein Assay Kit (TransGen Biotech, Beijing, China).

### Insects and Bioassays

The *C. bowringi* standard used in this study was donated by the Institute of Plant Protection (IPP), Chinese Academy of Agricultural Sciences (CAAS). *C. bowringi* insect eggs were incubated in a biochemical incubator at 25°C for 4–5 days. Fresh rape leaves were picked, washed, and placed on filter paper. The initially hatched *C. bowringi* larvae were gently inserted with a brush, and 5–6 larvae were placed in each Petri dish, which was placed in a biochemical incubator at 25°C (Liu et al., 2014). An appropriate amount of sterilized water was sprayed in the biochemical incubator in the morning and evening to maintain the air humidity. The leaves and filter paper in the Petri dish were changed every 24 h in the initial stage (6 days before incubation); the new filter paper was wetted, and it was ensured that the filter paper and rape leaves had no water droplets. After the initial stage, the Petri dish and the dead larvae were cleaned every day and the leaves and filter paper in the Petri dish were replaced every 12 h. The insect bodies were immersed in a pre-cooled saline solution. Sip1Ab, Cry8Ca, and a mixture of Sip1Ab and Cry8Ca in equal proportions were separately solubilized in 20 mM phosphate buffer before use in the bioassays. An analysis of the toxicity to *C. bowringi* was conducted on second instar larvae with fresh rape using the leaf-dip bioassay (Omer et al., 1993). The trays were incubated at 25 ± 2°C, 70 ± 5% RH, and a 10/8 h light/dark cycle, and mortality was scored at the second day. The quantitative bioassays were replicated at least three times using different concentrations of Sip1Ab, Cry8Ca, and a mixture of Sip1Ab and Cry8Ca. An insecticidal protein solution of PBS buffer was used as the control. The pET21b plasmid was used as the negative control. The LC_50_ value was measured with SPSS software. It is generally believed that the toxicity ratio of LC_50_ measured is expected to be 0.5–2.6. A value higher than 1.5 was considered synergistic, and less than 0.5 as antagonistic.

### Transmission Electron Microscope Observation

After starvation for 48 h, the *C. bowringi* larvae were fed the corresponding sensitive Bt proteins. Fresh and identical rapeseed leaves were selected. Protein samples were prepared and added with 0.1% detergent evenly smeared on both sides of the leaves with sterilized brushes. The leaves coated with protein samples were dried on the fresh-keeping film, and PBS buffer was applied as control. The concentration of Sip1Ab was 0.5 μg/ml. The sublethal worms and controls were dissected at 1, 2, and 3 days under microscopy. The midgut was immersed in PBS and diluted into 2.5% glutaraldehyde and fixed overnight at room temperature. The obtained midguts were prepared according to the method of transmission electron microscopy (TEM) sample preparation of Sinha et al. (2021). Uranium acetate peroxide dyeing was conducted for 10 min, lead acetate dyeing for 10 min, and a JEM-1230 (Joel, Tokyo, Japan) was used for electron microscopic observation.

### Brush Border Membrane Vesicle Extraction

To obtain the *C. bowringi* Brush Border Membrane Vesicles (BBMVs), the insect midguts were poured into a pre-cooled conical flask, then 30 ml Buffer A (300 mmol/L mannitol, 5 mmol/L EGTA, 17 mmol/L Tris, and pH 7.5) was added. The mixture was subsequently ground on ice and placed on ice for 15 min after 30 ml MgCl_2_ was added to the conical flask. Centrifugation (4,000 rpm) was conducted for 15 min. The supernatant was poured into a conical flask, and 15 ml MgCl_2_ was added. The supernatant was placed on ice for 15 min and centrifuged at 4,000 rpm for 15 min. The supernatant was decanted into 50 ml centrifuge tube and centrifuged at 10,000 rpm for 30 min. The precipitate was dissolved in 1 ml Buffer B (150 mmol/L NaCl, 5 mmol/L EGTA, 20 mmol/L Tris, 1% CHAPS, and pH7.5). The total BBMV protein was quantified with a Bradford Protein Assay Kit (Merck, NJ, United States).

### Ligand-Blotting Procedure

Proteins were biotinylated using the Pierce™ Cell Surface Protein Biotinylation and Isolation Kit (Thermo Fisher, MA, United States). Various concentrations of biotinylated Sip (NHS-Biotin-Sip) were added to BBMV in a range 25–100 nM at intervals of 25 nM. Additionally, 100 nM biotinylated Sip was separately added with a 10-, 15-, and 20-fold excess of unmarked Cry8Ca into the BBMV binding system. For the competitive binding assays, 0.1% BSA in PBS was added to all binding systems to make them up to 100 μl. All the systems were incubated for 1 h at room temperature. Unbound toxin was removed by centrifugation (10 min at 18,000 rpm), and the pellets were washed twice with PBS (pH 7.6, 0.1% BSA). The precipitate was suspended in 10 μl 1× SDS loading buffer, loaded on an SDS-PAGE gel, and electrotransferred to a PVDF membrane (GE Healthcare, MA, United States). The PVDF membrane was then incubated with Streptavidin-HRP (1:3000 dilutions) for 1 h at room temperature, followed by three wash cycles with PBST, 15 min per cycle. Binding was visualized using an ECL chemiluminescent kit (Thermo Scientific, MA, Untied States).

## Results

### Gene Cloning

**FIGURE 1:**
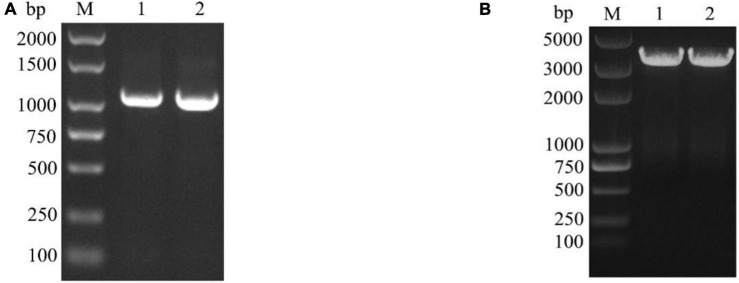
Verification of the *sip1Ab* and *cry8Ca* by PCR. **(A)** M: DL2000 DNA Marker, 1 and 2: the amplified *sip1Ab* from QZL38 genome with sipF and sipR primers. **(B)** M: DL5000 DNA Marker, 1 and 2: the amplified *cry8Ca* from QZL38 genome with cry8CaF and cry8CaR primers.

The designed full-length primers sipF/sipR and cry8CaF/cry8CaR were used to amplify the *sip1Ab* and *cry8Ca* by PCR. The amplified fragments of a size of 1,044 and 3,522 bp were consistent with the expected fragment size. The agarose electrophoresis results are shown in the figure below (Figure 1).

### Expression and Purification of Sip1Ab and Cry8Ca

**FIGURE 2:**
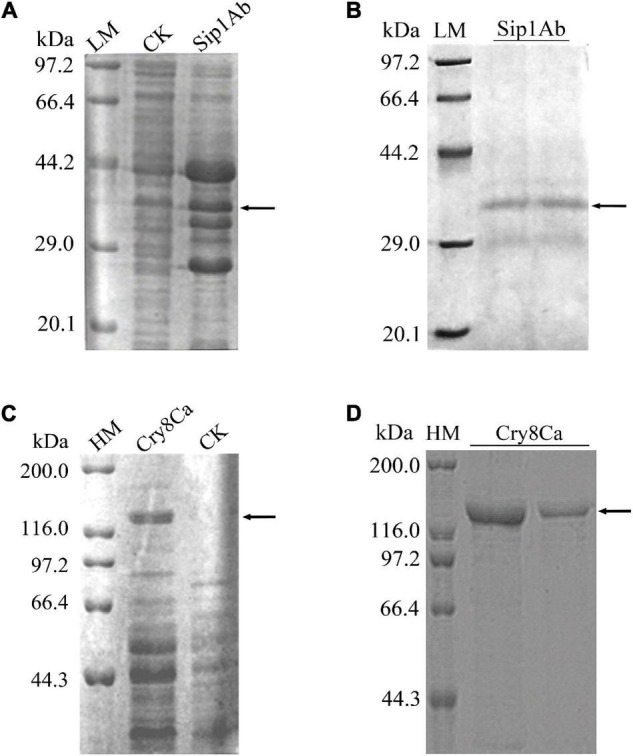
Expression and purification of Sip1Ab and Cry8Ca. LM: Protein Molecular Weight Marker (Low) (Takara Biomedical Technology Co., Ltd., Beijing, China), HM: Protein Molecular Weight Marker (High) (Takara Biomedical Technology Co., Ltd., Beijing, China), CK: whole protein of pET21b-BL21 (DE3). Samples were subjected to SDS-PAGE followed by Coomassie Blue staining. **(A)** Expression of soluble Sip1Ab in *E. coli*. **(B)** The purified Sip1Ab. **(C)** Expression of soluble Cry8Ca in *E. coli*. **(D)** The purified Cry8Ca.

Expression from recombinant plasmids *sip1Ab*-pET21b and *cry8Ca*-pET21b was induced by IPTG. With SDS-PAGE, the expressed products were shown to include a recombinant protein at approximately 37 kDa (Figure 2A) and 130 kDa (Figure 2C), indicating that both proteins were successfully expressed in *E. coli* BL21 (DE3). Subsequently, the His-tagged recombinant proteins were purified from the crude extract using Ni-NTA agarose affinity chromatography (GE Healthcare, MA, United States) (Figures 2B,D).

### Bioassays

**FIGURE 3:**
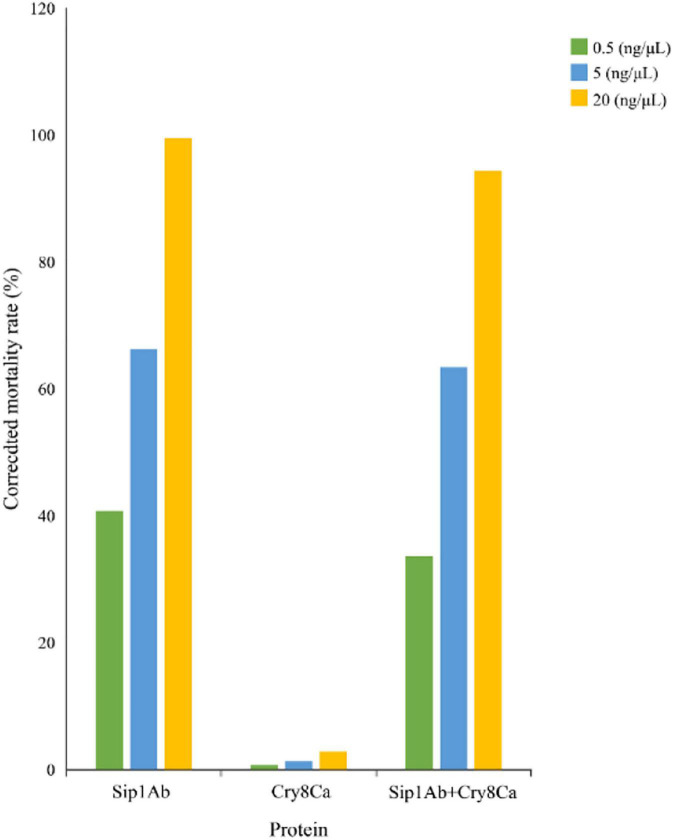
Qualitative bioassay results of Sip1Ab and Cry8Ca against *Colaphellus bowringi*.

The expression from recombinant strains was induced by IPTG. In the qualitative bioassays for the insecticidal activity of the proteins, second-instar *C. bowringi* were used as the test insects, and in that of the protein expressed in *E. coli*, pET21b was used as the negative control. The protein concentrations for bioassay were 0.5, 5, and 20 mg/ml. Forty-eight insects were tested for each protein, and the death of the insects was assessed 48 h later. The experiment was repeated three times. The test results are shown in Figure 3.

The insecticidal activity of Cry8Ca and Sip1Ab is shown in Table 2. The results showed that the insecticidal activity of a mixture of Cry8Ca and Sip1Ab (1:1) showed no significant difference from that of Sip1Ab.

**TABLE 2:** The bioassay results of Sip1Ab and Cry8Ca proteins against the *Colaphellus bowringi*.

Sample	LC_50 (_μ g ml^–1^)	95% confidence interval (μ g ml^–1^)
Sip1Ab	1.067	0.672–1.158
Cry8Ca	>200	–
Sip1Ab + Cry8Ca	1.462	0.855–1.361

### Observation of Midgut Histopathological Changes

#### Midgut Microvilli

**FIGURE 4:**
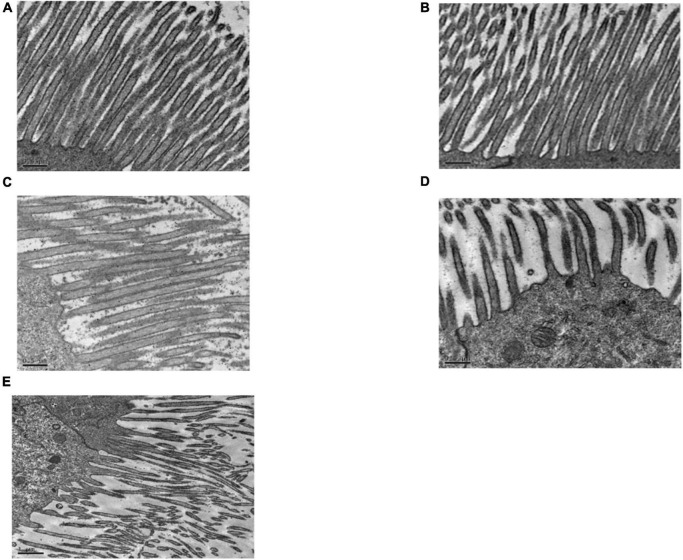
Pathological effects of Sip1Ab and Cry8Ca on midgut microvilli of larvae of *Colaphellus bowringi* **(A)** Control group fed PBS for 3 days, **(B)** Cry8Ca treatment for 3 days, and **(C–E)**: 1, 2, and 3 days after treatment with Sip1Ab, respectively.

Figure 4A shows that the midgut microvilli of healthy *C. bowringi* were dense, slender, and well-distributed and the membrane structure was normal. One day after treatment with Sip1Ab, the microvilli swelled and shed slightly and the structure was sparse (Figure 4C). After treatment with Sip1Ab for 2 days, a number of microvilli were exfoliated, and many vesicle structures were formed (Figure 4D). After 3 days of treatment with Sip, the microvilli were almost shed and disordered (Figure 4E). No significant change was seen in the group treated with Cry8Ca (Figure 4B).

#### Midgut Intercellular Space

**FIGURE 5:**
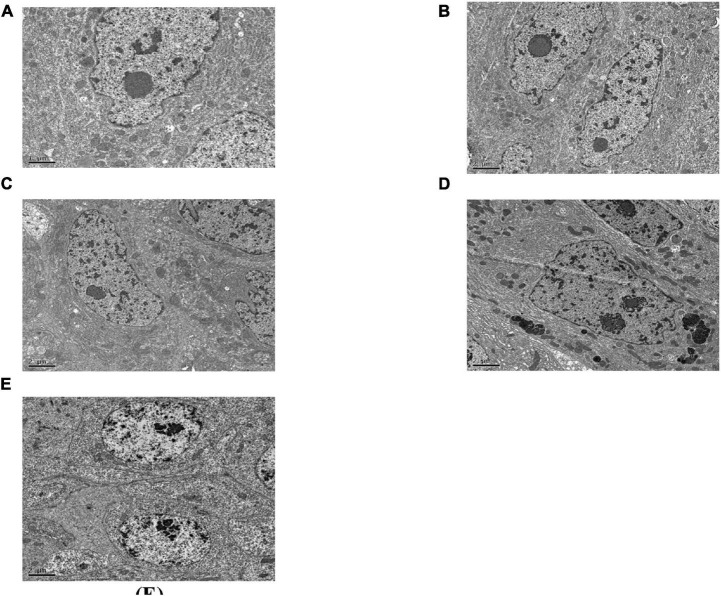
Pathological effects of the Sip1Ab and Cry8Ca on midgut space of larvae of *Colaphellus bowringi* **(A)** Control group fed PBS for 3 days, **(B)** Cry8Ca treatment for 3 days, and **(C–E)**: 1, 2, and 3 days after treatment with Sip1Ab, respectively.

As can be seen in Figure 5, the gap between the two cells in the midgut of the healthy *C. bowringi* was very small, with sporadic vesicles (Figure 5A). After 3 days of treatment, the gap between the two cells in the midgut appeared to be unaffected by Cry8Ca (Figure 5B). After 1 day of treatment with Sip1Ab, the gap between the two cells in the midgut increased significantly, and the slight packing structure increased (Figure 5C). During 2–3 days of treatment with Sip1Ab, not only did the gap between the two cells in the midgut increase significantly, but also the break-up and separation of cell membranes appeared (Figures 5D,E).

### Extraction of Brush Border Membrane Vesicles

**FIGURE 6:**
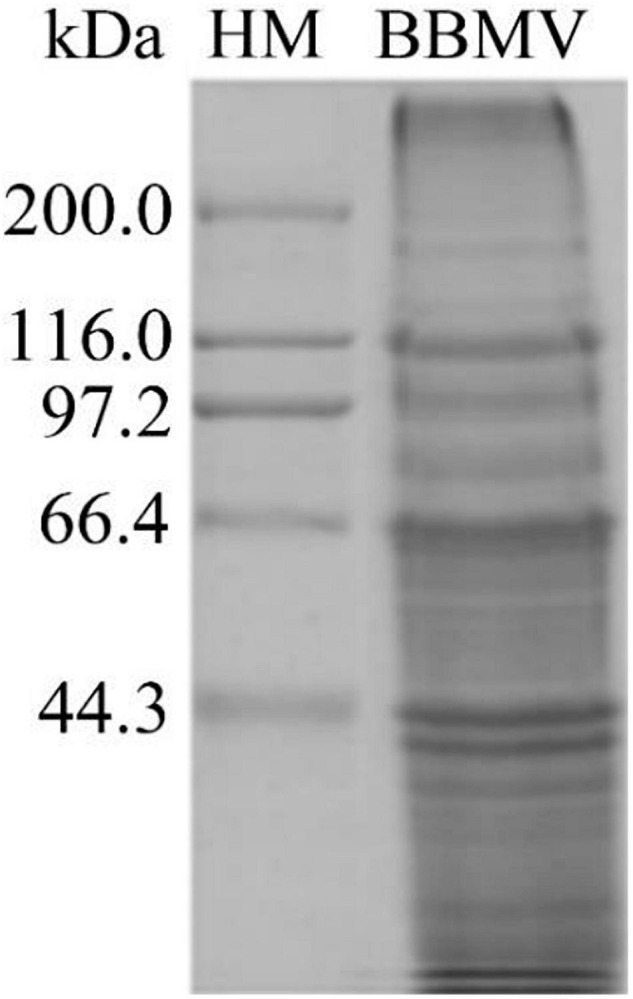
Proteins in *C. bowringi* BBMV were separated by SDS-PAGE. HM: Protein Molecular Weight Marker (High).

About 5 g of midgut tissue was extracted from around 1,500 *C. bowringi* fifth instar larvae. BBMV was extracted from the *C. bowringi* midgut (Figure 6). The protein concentration was determined by the Bradford method.

### Ligand Blotting

**FIGURE 7:**
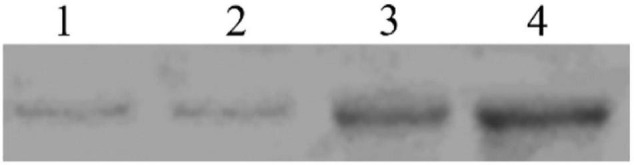
Determination of saturated binding between biotinylated Sip toxin and *C. bowringi* BBMV. 1–4: 25, 50, 75, and 100 nM biotinylated Sip, respectively.

In the binding test, the binding of biotinylated Sip1Ab to *C. bowringi* BBMV was tested *in vitro*. The ligand-blotting analysis demonstrated the binding of biotin labeled toxin (Sip1Ab) to protein in the *C. bowringi* BBMV (Figure 7). The binding activity of Sip1Ab could be clearly observed at a concentration of 100 nM.

**FIGURE 8:**
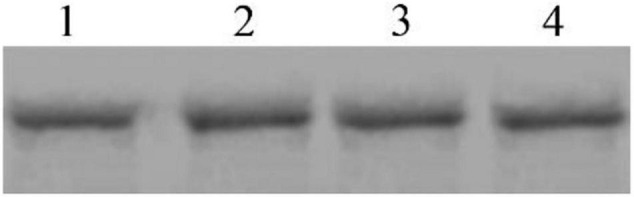
Competitive determination of Cry8Ca between biotinylated Sip binding in *C. bowringi* BBMV. 1: 100 nM biotinylated Sip, 2–4: Biotinylated Sip + Cry8Ca in 10-, 15-, and 20-fold excess, respectively.

To determine whether Cry8Ca exhibits competition during interactions between Sip1Ab and *C. bowringi* BBMV, a mixture of these two proteins was added to the BBMV binding system. As shown in Figure 8, excess Cry8Ca up to 20× was unable to compete with the binding of Sip1Ab to *C. bowringi* BBMV, suggesting that this could be a unique binding protein for Sip1Ab toxin.

## Discussion

*Colaphellus bowringi* is a common agricultural pest which occurs in most parts of China, and mainly damages cruciferous vegetables such as the *Brassica rapa* Chinensis Group, *Raphanus sativus*, *Brassica oleracea* var. *botrytis*, *B. rapa* var. *botrytis*, and so on (Zhu et al., 2019). A series of studies has shown that Sip has high insecticidal activity against coleopteran pests such as *L. decemlineata* and *C. bowringi* (Donovan et al., 2006; Sha et al., 2018; Wang et al., 2020). This observation provides a foundation for the discovery of novel genes of Bt and for expanding its insecticidal spectrum. However, to date, *sip* has only been studied with mutants and truncated genes. Histopathological changes in the midgut due to poisoning by Sip toxin, and studies on the heterologous competition between Sip1Ab and another Bt toxin, have not yet been reported. So, TEM was used to observe the midgut tissue. Additionally, corresponding competition assays were carried out to gain more insight into the insecticidal mechanism of Sip and the biocontrol of *C. bowringi*. Extensive research has shown that Cry8Ca was reported to have high insecticidal activity against coleopteran beetles (Jiang et al., 2017). Since Bt strain QZL38 contained *sip1Ab* and *cry8Ca*, it was speculated that there was a linkage between these two toxins vis a vis toxicity to *C. bowringi*. According to the results of bioactivity assay, Sip1Ab is almost certainly has high insecticidal activity, while Cry8Ca may have no insecticidal activity against *C. bowringi*. Furthermore, compared with a mixture of Sip1Ab and Cry8Ca, no significant difference in the LC_50_ of Sip1Ab was noted. A possible explanation is that no synergistic or antagonistic effect between these two toxins exists. Histopathological changes of the midgut were observed, further verifying the bioassay results. This inconsistency may be due to the different receptors for Sip1Ab and Cry8Ca in the midgut. In future investigations, on the one hand, it is possible that Sip1Ab and Cry8Ca have synergistic or antagonistic effects on the control of other coleopteran pests. For example, a study might be carried out on the basis of the known insecticidal activity of Sip against *L. decemlineata*. On the other hand, to develop a full picture of the correlation between Sip1Ab and Cry8Ca, additional studies could establish the basis of the insecticidal activity of Cry8Ca against *A. corpulenta* and *A. exoleta* Fald.

The midgut receptor was initially found to be a protein that could bind to membrane surface glycoproteins (Mayerson and Hall, 1986). Some studies have shown that a high affinity binding site exists between Bt toxins and BBMV in the larval midgut, and the corresponding effect was positively associated with the concentration of the toxins (Höfte and Whiteley, 1989). The findings of this study suggested that Sip interacted with BBMV. Analogous to the insecticidal mechanism of the Cry toxins, it is possible that the Sip toxins must be hydrolyzed by midgut proteases into active fragments to function. Further work should focus on determining the receptors for Sip1Ab in the BBMV *via* pull-down or yeast two hybrids. Nevertheless, Sip1Ab and Cry8Ca did not compete with each other in the combination assays. A possible explanation for this might be that Sip1Ab and Cry8Ca may have synergistic or antagonistic effects in different pests. Therefore, Sip1Ab may be beneficial for the avoidance of the risk of cross-resistance of insects to Cry8Ca toxins. Moreover, as has been observed in many laboratory and field pests, pyramiding with another toxin with a different mode of action and binding site would be desirable to circumvent the development of resistance to an insecticidal protein, and Sip toxins may be candidates for this.

## Conclusion

In summary, while this study did not confirm that a specific receptor of Sip1Ab exists in the midgut, it did substantiate Sip1Ab has interactions with the BBMV. To the best of our knowledge, it is the first time pathological changes in the *C. bowringi* larvae midgut due to poisoning by Sip1Ab were observed using TEM. Additionally, the interaction between Sip1Ab and the BBMV in the midgut was confirmed, which has advanced our understanding of the specific insecticidal mechanism of Sip1Ab. It can also provide genetic resources for the control of coleopteran pests and shine new and important light in the field of crossing resistance improvement strategy design.

## Data Availability Statement

The datasets presented in this study can be found in online repositories. The names of the repository/repositories and accession number(s) can be found in the article/supplementary material.

## Author Contributions

DC, CX, and QF: conceptualization. DC, XL, RL, and HL: methodology. CX: data analysis. DC and QF: writing—original draft preparation. CX and HL: writing—review and editing. JG: supervision, project administration, and funding acquisition. All authors have read and agreed to the published version of the manuscript.

## Conflict of Interest

The authors declare that the research was conducted in the absence of any commercial or financial relationships that could be construed as a potential conflict of interest.

## Publisher’s Note

All claims expressed in this article are solely those of the authors and do not necessarily represent those of their affiliated organizations, or those of the publisher, the editors and the reviewers. Any product that may be evaluated in this article, or claim that may be made by its manufacturer, is not guaranteed or endorsed by the publisher.
